# Evaluation of the antitrypanosomal activity, cytotoxicity and phytochemistry of red Brazilian propolis

**DOI:** 10.1371/journal.pone.0313987

**Published:** 2024-11-19

**Authors:** Samyah Alanazi, Naif D. Alenzi

**Affiliations:** 1 Clinical Laboratory Sciences Department, College of Applied Medical Sciences, King Saud University, Riyad, Saudi Arabia; 2 Research and Laboratories Sector, National Drug and Cosmetic Control Laboratories (NDCCL), Saudi Food and Drug Authority, Riyadh, Saudi Arabia; Kwame Nkrumah University of Science and Technology Faculty of Pharmacy and Pharmaceutical Sciences, GHANA

## Abstract

Recently, the growth in the consumption of functional foods with potential nutritional and health benefits revealed rapid progress in phytochemical analysis to assure quality and profile the chemical composition. Bee propolis, a gummy exudate produced in beehives after harvesting from different plant species and showed to contain bioactive secondary metabolites with biological importance. The main goal of the current study is to profile the chemical composition of red propolis samples from the Brazilian stingless bee *Tetragonula biroi* for the first time using HPLC-UV-ELSD and NMR analysis for assignment of the abundant metabolites’ classes as well as extraction and isolation of the major compounds. Column chromatography and size exclusion chromatography were applied for the purification of the major compounds in red Brazilian propolis. Further, testing the antitrypanosomal and cytotoxic activities against *Trypanosoma brucei* and human leukemia cell lines (U937) was performed. A total of 29 secondary metabolites were identified as two anthocyanins, 6 flavonoids, 8 isoflavonoids, 10 phenolics, two phenolic acids, and one triterpenoid. Two phenolic compounds were purified and identified using 1D and 2D NMR analysis along with MS analysis as liquiritigenin and calycosin. Red Brazilian propolis FB-3 fraction showed the highest inhibitory activity against *T*. *brucei* at 1.6 μg/ml, compared to 12.4 μg/ml of the crude extract. The isolated compounds showed moderate activity with an MIC of 8.5 μg/ml for liquiritigenin and 8.7 μg/ml for calycosin. Moreover, FB-3 fraction and calycosin were showed the potent cytotoxic effect with IC_50_ = 45.1 and 35.8μg/ml, respectively compared to IC_50_ = 29.5 μg/ml of the standard diminazen. Hence, red Brazilian propolis is rich source of polyphenols with myriad biological importance. Propolis fractions and purified compounds showed moderate antiprotozoal activity and potent cytotoxic activity against human leukemia cell lines.

## Introduction

Recently, the growth of consumption of functional foods with both nutritional and health benefits led to a progress in analytical tools used for assuring their quality and profiling of its secondary metabolites [1]. Bees can produce several products including propolis, pollen, and bread with potential nutritional value and myriad of health benefits [1]. As a gummy resinous exudate produced in bee colony after harvesting resins from different plant species, bee propolis comprises several secondary metabolites with potential health effects [2]. The harvested resins are mixed with bees wax in bee hives to produce propolis with different chemical composition [3]. Bee propolis is formed during sterilization of beehives due to its antimicrobial properties comes from the bioactive metabolites from plant species to ensure a healthy hive environment [4]. The phytochemical composition of bee propolis changed according to several factors including geographical, and botanical source, and the bee species [5].

Different types of Brazilian propolis among which green Brazilian propolis (derived from *Baccharis dracunculifolia*), red Brazilian propolis (*Dalbergia ecastophyllum*) were identified [6]. Propolis chemical composition is related to both its botanical source and environmental conditions [7]. The main botanical sources of Brazilian red propolis are *Dalbergia ecastaphyllum*, a rich source of isoflavonoids, and *Symphonia globulifera*, a rich source of polyprenylated benzophenones (guttiferone E and oblongifolin B) and triterpenoids (β-amyrin and glutinol) [8]. Brazilian red propolis stands out for its health benefits, which are attributed to a phenolic-rich composition, mainly isoflavonoids such as formononetin, vestitol, and neovestivol with strong antioxidant, anti-inflammatory, and antimicrobial properties [7].

Following extensive study of green propolis from Brazil, a new kind of propolis has begun to attract attention, namely, red propolis from Brazil. Initially acquired in Maceio City (Alagoas state) in north-east Brazil, the product consists of compounds from the plants *Populus* sp. (poplar plant) and *Baccharis dracunculifolia* [9]. The discovery and investigation of a range of types of red propolis are anticipated to provide further knowledge about the product [10]. Compounds from the plant *Dalbergia ecastophyllum* were identified by Daugsch et al., [11] to be present in the two types of red propolis, especially flavonoids like rutin, liquiritigenin, daidzein, pinobanksin, luteolin, and isoliquiritigenin. In terms of its composition, this type of red propolis was not the same as red propolis initially established to contain materials from *Populus* sp. and *B*. *dracunculifolia*.

Several secondary metabolites were reported in bee propolis belonging to different classes including flavonoids, terpenoids, and phenolics [5]. *Tetragonula biroi* propolis was reported to contain triterpenoids such as isomangiferolic acid, 27-hydoxymangiferonic acid, and 27-hydroxyisomangiferolic acid [4]. Saudi propolis was previously examined and was rich in phenolic compounds such as fisetinidol and ferulic acid [2,12]. The chemical composition of Cuban red propolis and Brazilian red propolis was investigated using HPLC-DAD-MS/MS revealing the identification of isoliquiritigenin, liquiritigenin and naringenin, isoflavones, isoflavans, and pterocarpans [13]. Several pharmacological properties such as anti-inflammatory, immunomodulatory [14], antimicrobial, antioxidant [15], antitumor [16], antiulcer [17] and anti-diabetic activities [18], were reported for bee propolis. Propolis was used traditionally to treat colds, wounds, ulcers, rheumatism, sprains, and dental caries [19]. The *in vitro* cytotoxic activity of several flavonoid compounds isolated from the Brazilian red propolis methanol extract was investigated against six different cancer cell lines including murine colon 26-L5 carcinoma, murine B16-BL6 melanoma, murine Lewis lung carcinoma, human lung A549 adeno carcinoma, human cervix HeLa adenocarcinoma, and human HT-1080 fibrosarcoma cell lines revealing the activity of 7-hydroxy-6-methoxyflavanone against the six cancer cell lines [20].

The main goal of the current study was to profile red propolis chemical composition from the Brazilian stingless bee *Tetragonula biroi* using HPLC-UV-ELSD for the first time. Additionally, the major secondary metabolites in Brazilian propolis samples were isolated and purified using different chromatographic techniques. Moreover, the antitrypanosomal and cytotoxic activity of Brazilian propolis were tested against *T*. *brucei* and human leukemia cell line, respectively.

## Materials and methods

### Propolis collection and preparation

The propolis from the Brazilian stingless bee *Tetragonula biroi* Friese was collected from Maceio City (Alagoas state) in north-east Brazil (9°39′57″S 35°44′06″W) by Mr James Fearnley according to SISGEN guidelines. Raw propolis was collected in an airtight container and kept at 25°C for 24 h. Later, propolis chunks were shipped to Saudi Arabia in ice-cold conditions. Prior to extraction techniques, propolis was freed of impurities, such as pollen, wood, the dead remains of bees, etc., and fragmented manually using a mortar and pestle.

### Chemicals and reagents

Solvents including acetonitrile, methanol, ethyl acetate, water, formic acid (LC-MS-grade), Davisil grade 633 amorphous precipitated silica (pore size 60 A, mesh size 200–425 _m), Sephadex LH-20, *p*-anisaldehyde, vanillin, sulfuric acid, and the deuterated solvents chloroform-*d* (CDCl_3_) and dimethyl sulfoxide-*d6* (DMSO-d6) were obtained from Sigma-Aldrich (Gillingham, UK). Glass columns for column chromatography were obtained from Rotaflo, Fisher Scientific, (Loughborough, UK). TLC-grade silica gel (60H) and TLC silica gel 60 F_254_ precoated aluminum sheet and the HPLC-grade solvents ethyl acetate, methanol, acetonitrile, *n*-hexane, and absolute ethanol were purchased from Merck, Darmstadt, Germany. Alamar blue® BUF 012B (AbD Serotec®, Oxford, UK), HMI-9 medium (Invitrogen, Oxford, UK), RPMI-1640 (Lonza, Verviers, Belgium), L-glutamine (Life Tech, Paisley, UK), Penicillin/ Streptomycin (Life Tech, Paisley, UK), fetal bovine serum (FBS) (Sigma-Aldrich, Gillingham, UK), U937 cell cultures (obtained from ECACC, Porton Down, Salisbury, 9 of 13 UK), 96-well plates (Corning®, Sigma-Aldrich), and a plate reader (Perkin Elmer, Buckinghamshire, UK) were also obtained.

### Extraction and purification of propolis

#### Extraction

The extraction process was done as previously reported in literature with some modifications [4,21]. Propolis sample (40 g) was extracted with 150 mL of 70% ethanol three times with sonication at room temperature for 60 min. The combined extracts were evaporated under reduced pressure using a rotary evaporator, and the residue was weighed.

#### Purification

The purification of pure phenolic compounds was performed as previously reported [21]. The ethanolic extract of red Brazilian propolis (10.5 g) was subjected to silica column chromatography and elution was sequentially performed using a gradient profile of solvent systems *n*-hexane/ethyl acetate and ethyl acetate/methanol as described below. The obtained fractions were collected according to their similarities and further gathered via HPLC-UV-ELSD analysis based on similar chemical profiles. The total number of fractions generated was 29, and these were collected in vials with a volume of 50 ml. Fractions were collected according to chromatographic screening using TLC and with suitable solvent system. Further LC-MS and NMR-based profiling of the obtained fractions led to identification of the different components classes and allowed combination of fractions to get 9 collected fractions. Fraction (FB-3) (470 mg) obtained from column chromatography was subjected to more purification by size-exclusion chromatography, yielding 42 subfractions (FB-3-1 to FB-3-42), which led to acquisition of two isolated compounds (FB-3-10 and FB 3–14).

#### Materials for column chromatography

For column chromatography, silica gel 60 with a mesh size of 200–425 μm was used. The column was wet packed with approximately 50 g of silica slurry, and the solvent with the lowest polarity (i.e., *n*-hexane) was mixed before pouring and packing in a suitably sized glass column, e.g., (55 × 3 cm). The dried extract was then loaded on the column top and eluted with sequential performance using 200 mL of *n*-hexane, ethyl acetate, and methanol mixtures as follows: *n*-hexane/ethyl acetate (80:20), *n*-hexane/ethyl acetate (60:40), *n*-hexane/ethyl acetate (40:60), *n*-hexane/ethyl acetate (20:80), ethyl acetate and then ethyl acetate/methanol (80:20), ethyl acetate/methanol (60:40), ethyl acetate/methanol (40:60), and ethyl acetate/methanol (20:80).

HPLC-UV-ELSD was performed on an Agilent 1100 system (Agilent Technologies, Waldbronn, Germany) using a reverse-phase C18 column with water and acetonitrile as the mobile phase. The interpretation of the data was carried out using Clarity software (Data Apex). For Size-Exclusion Chromatography, Sephadex LH 20 slurry was prepared by the overnight suspension of the stationary phase in 50:50 dichloromethane/methanol for nonpolar fractions and in 100% methanol for polar fractions. A glass column (2 × 100 cm) was packed with the formed sephadex LH 20 slurry and covered with cotton wool. After loading the sample, elution was performed with 100% MeOH in an isocratic manner, and fractions were collected in small vials of around 1 mL.

#### Structure elucidation

*Nuclear Magnetic Resonance*. NMR acquisition was performed by a JEOL (JNM LA400) spectrometer (400 MHz) at SIPBS and a Bruker Avance 300 (400 MHz) spectrometer with tetramethylsilane (TMS) as the internal standard at King Saud University, Riyad, Saudi Arabia. One- and two-dimensional experiments were carried out to identify the chemical structure of the compounds present in the fractions. The compounds were prepared in deuterated solvents, such as CDCl_3_ and DMSO-*d6*, based on their solubilities. Then, 500–600 μL of a suitable solvent was used for the dissolution of 10 mg of every sample, which were then poured into typical NMR tubes (5 ×178 mm) to a depth of around 4 cm. The NMR spectral data were obtained using MestReNova software 8.1.2 (Mestrelab Research, A Coruña, Spain), and ChemBioDraw Ultra, Version 14 (PerkinElmer, Yokohama, Japan), was employed for illustrating figures of the structures of the isolated compounds.

*LC-MS analysis*. Like NMR, mass spectrometry (MS) provides structural data (molecular weight and molecular formula) of the examined compounds. Around 1 mg/mL of the crude and purified compounds were prepared separately for chemical profiling and molecular mass determination by LC-MS using a Dionex 3000 HPLC pump/Orbitrap mass spectrometer (Thermo Fisher Scientific, Bremen, Germany) at King Saud University, Riyad, Saudi Arabia. A reverse-phase 5 μm C18 column (4.6 ×150 mm) (Hypersil, Thermo) was used and eluted using a gradient at a flow rate of 0.3 mL/min with 0.1% v/v formic acid in water and 0.1% v/v formic acid in acetonitrile as the two solvents (A and B) making up the mobile phase. The ESI interface in negative ionization permitted the identification of [M-H]^-^. The spray voltages for the capillary and the cone were, respectively, -4.0 kV and 35 V. The flow rates of the sheath gas and auxiliary gas were, respectively, 50 and 15 arbitrary units. The ion transfer capillary had a temperature of 275°C, and m/z 100–1500 provided the full scan data. The sample data were acquired and processed with the Xcalibur software (Thermo Fisher Corporation, Hemel Hempstead, UK). The compounds were putatively annotated by comparing their MS data with the literature.

#### In vitro antitrypanosomal assay

An Alamar blue assay was carried out to evaluate the antitrypanosomal activity using a method described previously [4]. To perform the test, *T*. *brucei* S427 cells at a seeding density of 2 ×10^5^ cells and propolis samples (20 mg/mL in 100% DMSO) that were double-diluted to varying concentrations (0.19 to 200 μg/mL) were prepared using Hirumi’s Modified Iscove’s medium 9 (HMI-9) as a diluent. Then, 100 μL of each propolis sample was added to a 96-well plate, followed by the addition of a trypanosome suspension (100 μL) to each well and incubation for 48 h at 37°C in 5% CO_2_. After incubation, resazurin dye was added and incubated for a further 24 h under the same conditions. Following incubation, the fluorescence was recorded at Ex/EM: 544/620 nm using an FLUO star Optima (BMG Labtech, Offenburg, Germany).

#### Evaluation of cytotoxic activity using cell viability assay

RPMI 1640 medium was used for the culturing and subculturing of human leukemia cell lines (U937). The medium was supplemented with penicillin and streptomycin (1% v/v), L-glutamine (1% v/v), and FCS (5% v/v), and U937 cells were cultured in desirable conditions, viz. a temperature of -37°C, 100% humidity, and 5% CO_2_. To perform the cell viability assay, 100 μL of U937 cell suspension (containing 1×10^5^ cells/mL) was plated in each well and incubated for 24 h. Post incubation, cells were treated with crude and purified propolis samples prepared in varying concentrations (1.56–200 μg/mL) and incubated further for 24 hr. DMSO was added and served as a positive control (to kill the cells completely); the negative control was the cells with medium. Following incubation, the resazurin indicator (10% Alamar blue) was loaded into the wells and incubated for an additional 24 hr. The fluorescence of the plate was read using a Wallac Victor 2 microplate reader (Ex/EM: 560/590 nm), and cell viability was calculated and expressed as mean inhibitory concentration (IC_50_) values.

#### Statistical analysis

Antitrypanosomal and cytotoxic activities were expressed as means and standard errors. A paired t-test analysis was performed to determine the significance in the mean values of the anticancer and antitrypanosomal activities between the crude and isolated compounds. *p* < 0.05 was considered statistically significant. Statistical significance was determined using an unpaired two-tailed Student’s t-test comparing IC_50_ value of the resistant strain with that of the same sample for the control strain. Pentamidine and Diminazen aceturate, both are known trypanocides.

## Results

### Screening of chemical profile of red Brazilian propolis using LC-MS and NMR

LC-MS profiling of the ethanolic extract of red Brazilian propolis revealed the identification of 29 peaks among which two anthocyanins, six flavonoids, eight isoflavonoids, ten phenolics, two phenolic acids, and one triterpenoid were identified (**Table 1**). The Chemical profiling was carried out using many instrumental methods including high performance liquid chromatography (HPLC) coupled to different detectors such as an evaporative light scattering detector (ELSD), **S1A Fig in S1 File**, ultraviolet detection (UV), and high-resolution mass spectrometry (HR-MS) in addition to NMR spectroscopy. To identify the primary features of the constituents, preliminary NMR analysis was conducted on a 10 mg sample of the extract (**S1B Fig in S1 File**), followed by application of LC-MS for the purpose of LC-MS profiling, as indicated in **Table 1** and **S1C Fig in S1 File**. HPLC-UV-ELSD of the crude extract sample showed the presence of compounds with UV-absorbing activity, that could be flavonoids and phenolic compounds. Compounds without chromophores like terpenoids were detected but with low intensities (**S1A Fig in S1 File**). Considerable complexity was displayed by the LC-MS chromatogram of the crude extract, with numerous peaks that were intense. As indicated in **Table 1** and **S1C Fig in S1 File**, the crude extract largely consisted of flavonoids, phenolics and terpenoids, according to the results of LC-MS analysis. The prevalence of flavonoids and phenolics was also confirmed by the ^1^H NMR spectra (**S1B Fig in S1 File**) which contained many signals for aromatic protons.

**Table 1:** The LC-MS profiling for ethanolic extract of red Brazilian propolis when analyzed by reversed phase LC-MS in negative ion mode.

Peak No.	RT (min)	[M-H]^-^	Chemical formula	Delta (ppm)	Intensity	Compound	Class	Reference
Anthocyanins								
3	10.26	271.06	C_15_H_11_O_5_	-0.578	E 6	Luteolinidin	Anthocyanins	[22]
6	10.93	331.08	C_17_H_15_O_7_	2.428	E 6	Malvidin	Anthocyanins	[23]
Flavonoids								
5	10.26	299.06	C_16_H_11_O_6_	-1.409	E 6	Kaempferide	Flavonoid	[24]
9	11.62	301.07	C_16_H_13_O_6_	2.387	E 6	Hesperetin	Flavonoid	[25]
13	13.77	255.07	C_15_H_11_O_4_	2.697	E 7	Liquiritigenin	Flavonoid	[26]
16	14.94	315.05	C_16_H_12_O_7_	4.457	E 6	Rhamnetin	Flavonoid	[24]
18	15.72	285.08	C_16_H_13_O_5_	2.782	E 7	Ponciretin	Flavonoid	
23	21.98	269.05	C_15_H_9_O_5_	1.648	E 6	Apigenin-7-olate	Flavonoid	
Isoflavonoids								
10	13.28	253.05	C_15_H_9_O_4_	2.719	E 6	Daidzein	Isoflavone	[7]
11	13.28	297.04	C_16_H_9_O_6_	2.891	E 6	5-hydroxypseudobaptigenin	Isoflavone	[27]
12	13.57	315.09	C_17_H_15_O_6_	3.074	E 7	Rosinidin	Isoflavone	[28]
14	14.36	283.06	C_16_H_11_O_5_	2.873	E 7	Calycosin	Isoflavone	[26]
15	14.66	269.08	C_16_H_13_O_4_	3.076	E 7	7-methoxy Apigeninidin	Isoflavone	[29]
20	18.75	267.07	C_16_H_11_O_4_	2.538	E 8	Formononetin	Isoflavone	[30]
21	19.63	239.07	C_15_H_11_O_3_	2.604	E 7	7,4′-dihydroxyflavylium	Isoflavone	[31]
26	33.21	281.05	C_16_H_9_O_5_	-0.166	E 6	Pseudobaptigenin	Isoflavone	[32]
Phenolics								
2	5.07	437.07	C_26_H_13_O_7_	3.441	E 6	Unknown phenolic	Phenolic	
7	11.62	153.02	C_7_H_5_O_4_	1.49	E 6	2,5-Dihydroxybenzoate	Phenolic	
8	11.62	273.08	C_15_H_13_O_5_	2.575	E 6	Phloretin	Phenolic	[33]
17	14.94	449.2	C_27_H_29_O_6_	4.003	E 6	Unknown phenolic	Phenolic	
19	18.46	271.1	C_16_H_15_O_4_	2.758	E 7	Unknown phenolic	Phenolic	
22	21.09	401.14	C_25_H_21_O_5_	0.805	E 6	Unknown phenolic	Phenolic	
24	27.05	299.09	C_17_H_15_O_5_	0.612	E 7	Unknown phenolic	Phenolic	
25	30.17	285.11	C_17_H_17_O_4_	1.149	E 7	Unknown phenolic	Phenolic	
27	36.23	507.24	C_30_H_35_O_7_	2.175	E 6	Unknown phenolic	Phenolic	
28	49.43	301.22	C_20_H_29_O_2_	0.951	E 6	Unknown phenolic	Phenolic	
Phenolic acid								
1	5.07	173.05	C_7_H_9_O_5_	0.713	E 6	Shikimic Acid	Phenolic acid	[34]
4	10.26	121.03	C_7_H_5_O_2_	-1.179	E 6	Benzoic acid	Phenolic acid	[35]
Triterpenoid								
29	51.19	469.33	C_30_H_45_O_4_	2.934	E 6	Gypsogenin	Triterpenoid	[36]

### Isolation and characterization of red Brazilian propolis pure compounds

**Fig 1:**
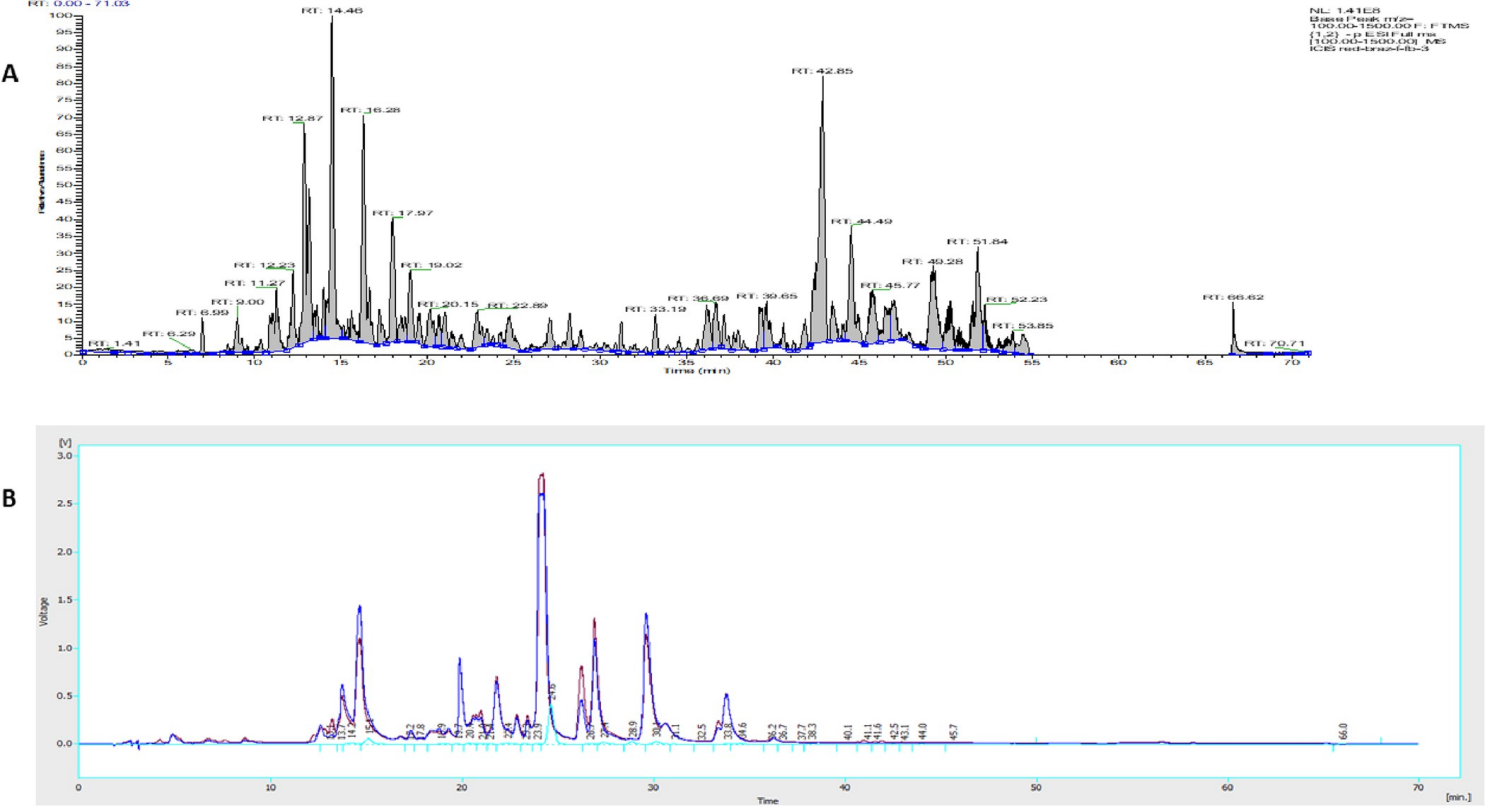
**A)** Chromatogram view of red Brazilian fraction FB-3 on the LC-MS in negative ion mode **B:** Chromatogram of the red Brazilian fraction (FB-3) on the ELSD-UV system; it is obvious that it consisted primarily of compounds that absorbed UV (blue trace), which could represent flavonoids and phenolics. Although compounds without chromophores such as terpenoids or fats were also identified (light blue trace) but their intensities were not high.

LC-MS (**Fig 1A**) and HPLC-UV-ELSD (**Fig 1B**) analysis highlighted the richest mixture of compounds was in fraction FB-3 with a varied composition (**S1 Table in S1 File**). Based on preliminary data, the compounds were most likely flavonoids, phenolics, and terpenoids. The ethanolic extract of red Brazilian propolis (10.5g) was subjected to column chromatography and elution was sequentially performed based on a gradient profile and collecting the fractions according to LC-MS and NMR profiles to yield 9 fractions (**S2 Table in S1 File**). Fraction FB-3 (470 mg) was subjected to size-exclusion chromatography to yield 42 sub-fractions (FB-3-1 to FB-3-42), which led to acquisition of two isolated compounds (**FB-3-10 (25 mg)** and **FB-3-14 (30 mg)**.

### Characterization of FB-3-10 as calycosin

Fraction FB-3-10 was obtained as a white powder appeared as a single spot-on silica TLC and elution with 40%, HE in EtOAc (R_*f*_ = 0.4) after spraying with *p*-anisaldehyde-sulphuric acid reagent and heating the chromatogram. The ESI-MS spectrum showed a molecular ion [M-H]—at *m/z* 283.06 corresponding to the molecular formula of C_16_H_11_O_5_ (**S2 Fig in S1 File**).

**Fig 2:**
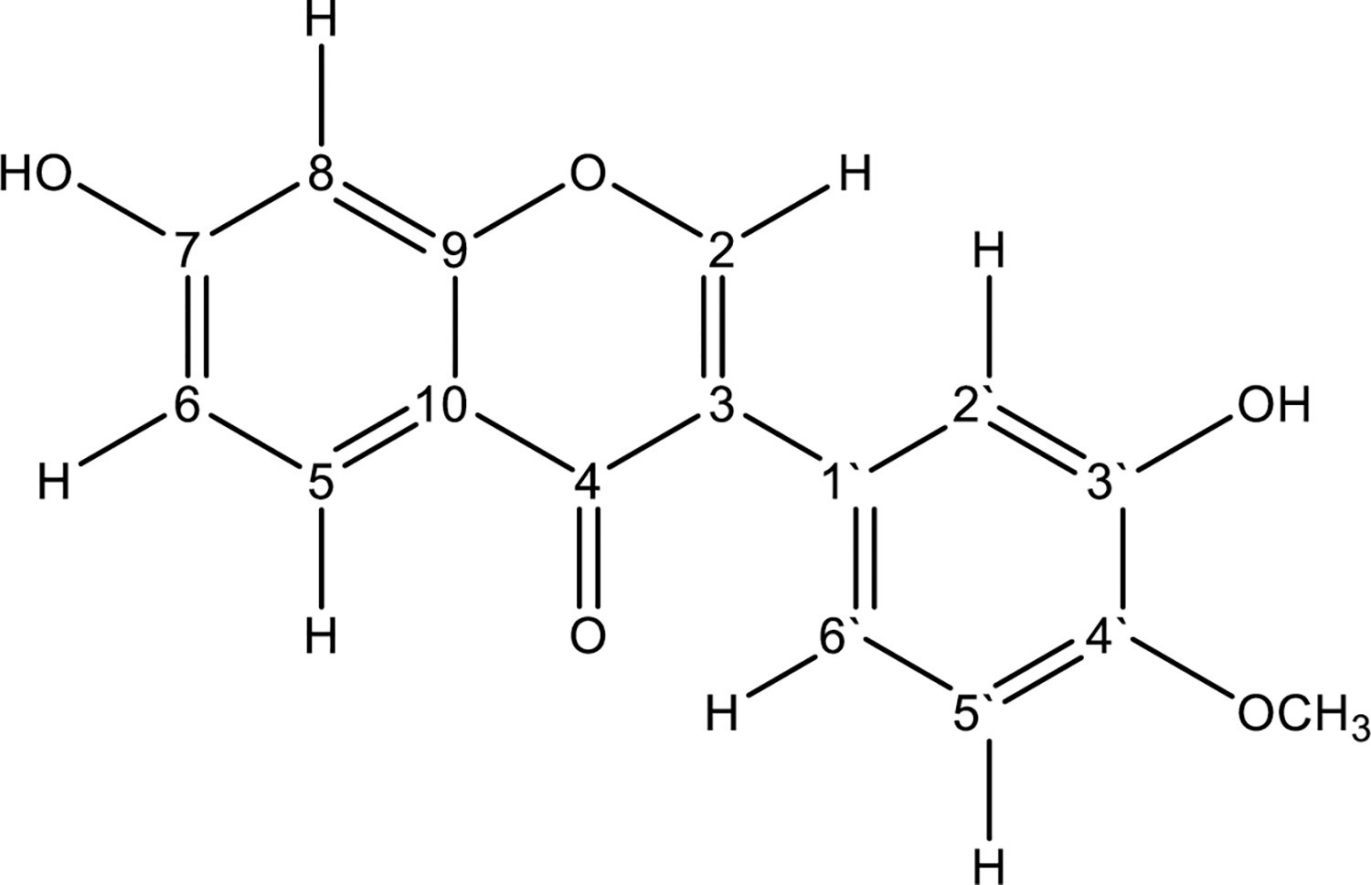
Structure of calycosin.

^1^HNMR (**S3 Fig in S1 File**, **Table 2**) showed a deshielded proton singlet at 8.29 ppm typical of the H-2 of an isoflavone. The proton spectrum of the compound showed two sets of aromatic ABX spin systems and that confirmed the presence of two trisubstituted benzene rings. The first set were at δ_H_ ppm 7.98 (d, *J* = 8.74), 6.93 (d, *J* = 2.26) and 6.87 (d, *J* = 2.24). The second set of the aromatic ABX protons were at 7.06 (d, *J* = 1.51), 6.96 and 6.96 (d, *J* = 2.01). Finally, a methoxy group was observed at 3.80 ppm. The ^13^C NMR spectrum (DEPT) (**S4 Fig in S1 File**) showed the presence of 16 carbon atoms made up of one carbonyl, one methoxy, 12 aromatic (including a phenolic and a methoxy substituted at 146.51 and 147.97 respectively) and two olefinic carbons conjugated to a carbonyl group. By comparing 1D spectral data and 2D NMR spectra including COSY (**S5 Fig in S1 File**), HSQC (**S6 Fig in S1 File**) and HMBC (**S7 Fig in S1 File**), with the reported literature, the structure was determined to be calycosin (**Fig 2**) [37].

**Table 2:** ^1^H (400 MHz) and ^13^C (100 MHz) NMR data of isolated compounds.

Calycosin (FB-3-10) in DMSO-d_6_			Liquiritigenin (FB-3-14) in CDCl_3_		
Position	^1^H (multiplicity), *J* (Hz)	^13^C (multiplicity)	Position	^1^H (multiplicity), *J* (Hz)	^13^C (multiplicity)
1			1		
2	8.29 (s)	153.5	2	5.44 (dd, *J =* 12.8, 2.8)	79.4
3		125.1	3a	2.63 (dd, *J =* 16.8, 3.01)	43.5
4		174.8	3b	3.11 (dd, *J =* 16.8, 12.9)	43.5
5	7.98 (d, *J* = 8.7)	127.7	4		175.0
6	6.93 (d, *J* = 2.2)	115.6	5	7.65 (d, *J =* 8.6)	127.7
7		162.9	6	6.51 (dd, *J =* 8.6, 2.2)	110.9
8	6.87 (d, *J* = 2.2)	102.5	7		163.5
9		157.8	8	6.33 (d, *J =* 2.2)	103.3
10		116.3	9		165.1
1’		123.8	10		113.9
2’	7.06 (d, *J* = 1.5)	116.9	1’		129.7
3’		146.5	2’	7.33 (d, *J =* 8.5)	115.5
4’		147.9	3’	6.80 (d, *J =* 8.5)	128.7
5’	6.96 (overlapped)	112.4	4’		158.1
6’	6.96 (d, *J* = 2.01)	120.1	5’	6.80 (d, *J =* 8.5)	128.8
7-OH	10.78 (s)		6’	7.33 (d, *J =* 8.5)	115.6
3`-OH	9.0 (s)				
4`-OCH3	3.80 (s, 3H)	56.16			

### Characterization of FB-3-14 as liquiritigenin

The fraction FB-3-14 purified from the ethanolic extract of red Brazilian propolis using CC and then SEC was obtained as a white powder and detected as single spot-on silica using 40%, HE in EtOAc as solvent system and *p*-anisaldehyde-sulphuric acid as spray reagent. The ESI-MS spectrum showed a molecular ion [M-H]—at *m/z* 255.07 corresponding to the molecular formula of C_15_H_11_O_4_ (**S8 Fig in S1 File**).

**Fig 3:**
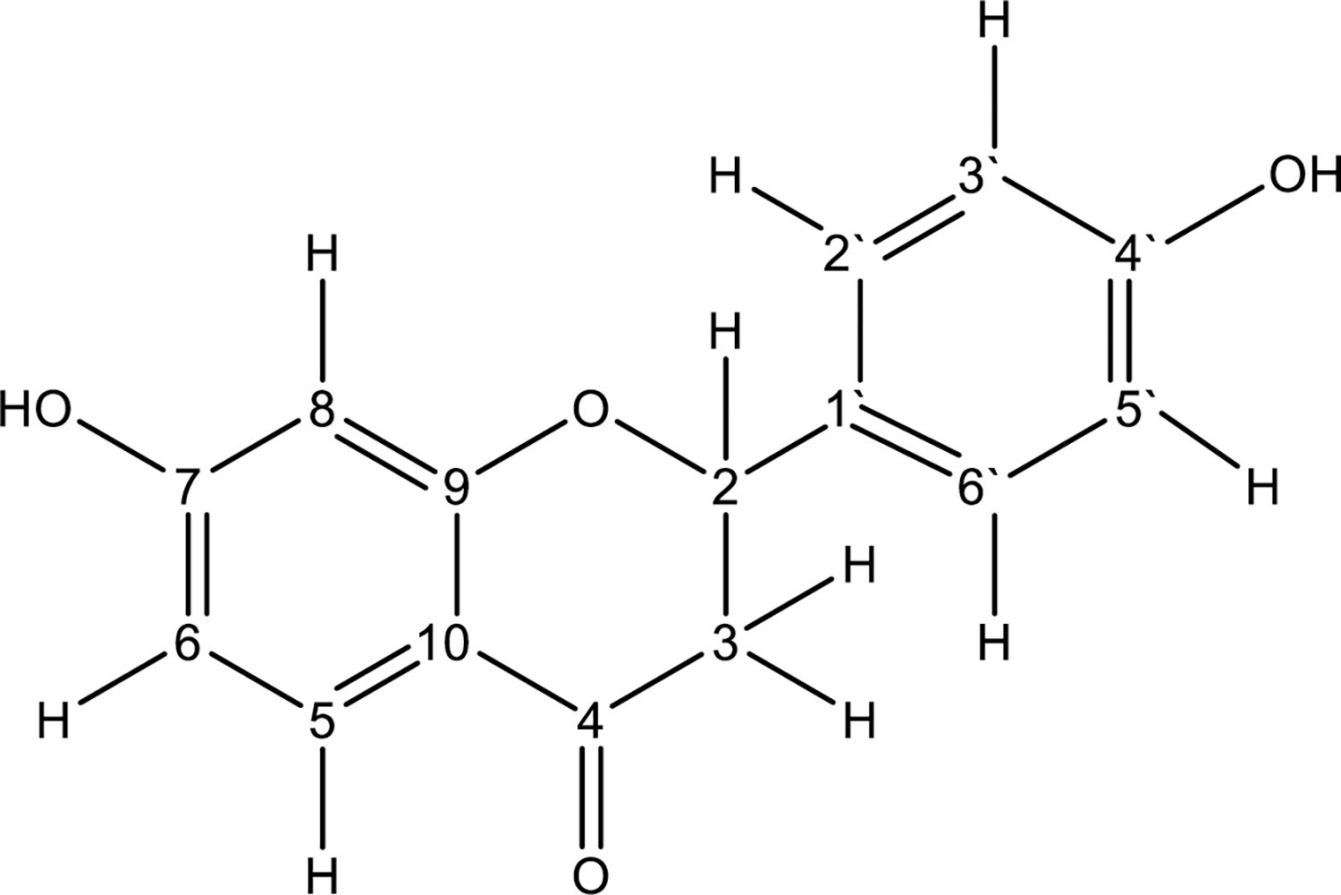
Structure of liquiritigenin.

In its ^1^H NMR (400 MHz) spectrum the compound (**S9 Fig in S1 File**, **Table 2**) showed a set of three aromatic protons with an ABX coupling at 7.88 (d, *J =* 8.6), 6.56 (dd, *J =* 8.6, 2.3) and 6.46 (d, *J =* 2.3) for a trisubstituted benzene ring and another four aromatic protons with an AA’BB’ coupling for a disubstituted benzene ring at 7.37 (d, *J =* 8.5), 7.38 (d, *J =* 8.5), 6.90 (d, *J =* 2.06) and 6.92 (d, *J =* 2.10). Three coupled aliphatic protons were observed at 5.42 (dd, *J =* 13.2, 2.8), 2.82 (dd, *J =* 16.8, 2.9) and 3.06 (dd, *J* = 16.8, 13.2). Their chemical shift values were indicative of proximity to a carbonyl or electron withdrawing group such as a benzene ring. The ^13^C spectrum (DEPT) (**S10 Fig in S1 File**) showed a total of 15 carbon signals made up of one carbonyl at 175.2, 12 aromatic carbons and two aliphatic carbons at 162.6 and 159.7. From the aforementioned chemical shift assignments for its proton and carbon and using 2D NMR spectral data including COSY (**S11 Fig in S1 File**), HSQC (**S12 Fig in S1 File**) and HMBC (**S13 and S14 Figs in S1 File**), and comparing with literature data, the structure was confirmed to be liquiritigenin (**Fig 3**) [38].

### Biological activities of red Brazilian propolis sample against trypanosome (*T*. *brucei* S427 strain)

Crude extract, fractions, and the isolated compounds (liquiritigenin and calycosin) extracted from the red Brazilian propolis sample were tested against *T*. *brucei*. Pentamidine and Diminazen were used as drug controls and their MIC values were 0.0048 and 0.0373 μg/ml respectively. The results from testing red Brazilian propolis extract and its components against *T*. *brucei* were listed in **Table 3**. The results showed a varying activity against *T*. *brucei* between tested samples. FB-3 fraction has a higher activity with MIC of 1.6 μg/ml (*p*> 0.05), than the crude extract which showed MIC of 12.4 μg/mL, and this was the most active fraction isolated in the present study. The pure compounds showed moderate activity with an MIC of 8.5 μg/ml for liquiritigenin and 8.7 μg/ml for calycosin (*p*> 0.05). Overall, crude, fractions and pure compounds were mainly moderately active against *T*. *brucei* S427 WT (**Table 3**).

**Table 3:** Drug sensitivity assay of red Brazillian propolis sample and its fractions on *T*. *brucei* S427 WT.

Propolis Sample	MIC(μg/ml) (Mean ±SD)	%RSD
Red Brazilian crude	12.4±0.62	5.02
FB-3 fraction	1.6±0.11*	6.50
Liquiritigenin	8.5±1.63*	19.13
Calycosin	8.7±0.52*	5.99
Pentamidine(μM)	0.0048±0.0004	7.6110
Diminazen(μM)	0.0374±0.0017	4.4221

* = *p*> 0.05.

### Cytotoxic activity of red Brazilian propolis sample against leukemia cell line U937 cells

Crude extract, fractions, and the isolated compounds (liquiritigenin and calycosin) extracted from the red Brazilian propolis sample were tested leukemia cell line U937 cells. Pentamidine and Diminazen were used as drug controls and IC_50_ values at 13.32 μg /mL and 29.58 μg/mL, respectively. The results from testing red Brazilian propolis extract and its components against leukemia cell line U937 cells were listed in **Table 4**. It is worth noting that the crude extract as well as liquiritigenin samples gave increased cell viability (> 100 μg /mL) while FB-3 fraction and calycosin were showed a close toxicity result to diminazene as its minimum IC_50_ value > 30 μg/mL (*p*> 0.05). Pentamidine and Diminazene gave the lowest IC_50_ values at 13.32 μg /mL and 29.58 μg/mL, respectively.

**Table 4:** Cytotoxicity assay of red Brazilian propolis sample and its fractions on U937 cells.

**Propolis Sample**	**IC**_**50**_ **(μg/ml) (Mean ±SD)**	**%RSD**
Red Brazilian crude	107.9±11.20	10.38
FB-3 fraction	45.1±3.75*	8.33
Liquiritigenin	92.5±5.43	5.86
Calycosin	35.8±6.99*	19.5
Pentamidine(μM)	13.3167±1.0148	7.6202
Diminazen(μM)	29.5767±2.1704	7.3381

* = p> 0.05.

## Discussion

Recently, several researches on propolis derived from different geographical regions, including Europe, Asia, North America and South America, particularly Brazil [39,40]. In the case of Asia, comprehensive investigations [41] have focused on propolis from China [42], Japan [43], Taiwan [44], Nepal [45] and Myanmar [46]. The chemical composition of propolis is variable even in the case of propolis from the same geographical area [20]. In the present study, the raw propolis samples from Brazil were extracted and purified using successive chromatographic techniques to facilitate identification of the chemical composition of propolis samples. Two phenolic compounds were isolated from the ethanol extract of red propolis from Brazil and identified as calycosin and liquiritigenin. The isolation of isoflavonoids in Brazilian propolis along with flavonoids was in accordance with previous study on red propolis from Cuba produced mainly from the resin of *D*. *ecastophyllum* which contained the isoflavonoid medicarpin and liquiritigenin [47]. Moreover, liquiritigenin was previously identified in Cuban red propolis, Brazilian red propolis, and *D*. *ecastophyllum* exudates using HPLC-DAD-MS/MS [13]. Additionally, calycosin and liquiritigenin were isolated from red Nigerian propolis [35]. Recently, calycosin along with other 14 flavonoids were tentatively identified in Brazilian Amazon red propolis using LC-MS/MS [30]. To better assessment of the biological importance of Brazilian red propolis, the two isolated metabolites (calycosin and liquiritigenin) were tested for their biological activities. Testing the red Brazilian propolis sample for biological activities against trypanosome revealed that the FB-3 fraction showed the higher activity with MIC of 1.6 μg/ml than the crude extract which showed MIC of 12.4 μg/ml and can be considered with a moderate to high activity against *T*. *brucei* S427 WT. Moreover, calycosin and liquiritigenin showed nearly the same activity toward trypanosome with MIC 8.7 and 8.5 μg/ml, respectively. The results were in accordance with the previous study done by Omar et. al., which tested the antitrypanosomal activity of red Nigerian propolis [35].

Investigation of the cytotoxic activity using the cell viability test revealed inhibitory activity of the FB-3 fraction may result from cell toxicity in contrast to the crude sample which was not cytotoxic up to 100 μg/ml. Moreover, calycosin showed the higher cytotoxic activity among the tested samples with IC_50_ of 35.8 μg/ml which revealed its potent activity against leukemia cell line U937 cells. Li et al., 2008 studied the *in vitro* cytotoxicity of flavonoids isolated from red Brazilian propolis against six cancer cell line [20]. Results revealed that, 7-hydroxy-6-methoxyflavanone exhibited the most potent activity against murine B16-BL6 melanoma (IC_50_, 6.66 μM), murine LLC Lewis lung carcinoma (IC_50_, 9.29 μM), human lung A549 adenocarcinoma (IC_50_, 8.63 μM), and human HT-1080 fibrosarcoma (IC_50_, 7.94 μM) cancer cell lines, and mucronulatol against LLC (IC_50_, 8.38 μM) and A549 (IC_50_, 9.9 μM) cancer cell lines [48]. The antimicrobial, antiparasitic, and cytotoxic effects of extracts of red propolis from different regions of Brazil, obtained by ethanolic extraction was studied revealing antimicrobial activity against *Staphylococcus aureus*, antiparasitic activity against *Trypanosoma cruzi*, and cytotoxic effect against all four cancer cell lines tested (HL-60, HCT-116, OVCAR-8, and SF-295), indicating that red propolis extracts have great cytotoxic potential [49].

In order to standardize propolis extract, the chemical composition and biological properties are important to assess propolis quality [4]. Propolis has been reported to consist of over 300 compounds, but not all compounds are related to biological effects [9]. Under these circumstances, achievement of standardization of propolis samples from different geographical areas with different biological effects has not been possible so far [9]. To link a specific chemical type of propolis to a specific biological effect, additional study is required in order to accomplish standardization of propolis types [5]. Given the considerable complexity of the mixture produced by the various compounds present in propolis crude extracts, it is improbable to acquire a single pure compound from the crude extract through with just one separation method [2]. Consequently, the crude extract frequently must be fractionated into several different fractions with polarities or molecular sizes that do not differ much [4]. However, attention must be paid to the fact that detection of fractions with the compound in low concentration or detection of activity in bioassays in the case of bioassay-based isolation processes may fail if the number of fractions produced is too high, as this will result in the spreading of the compound in question across too many fractions [50].

## Conclusion

Phytochemical profiling and isolation of the major secondary metabolites in red Brazilian propolis and studying the antitrypanosomal and cytotoxic activities of the crude extract, fraction and isolated compounds was introduced for the first time. LC-MS, NMR tools were used for profiling of metabolites classes abundant in red Brazilian propolis. Further purification with CC and SEC led to the separation of two isolated compounds belonging to flavonoid and isoflavonoid and identified as liquiritigenin and calycosin. Biological investigation revealed that, FB-3 fraction showed higher activity with MIC of 1.6 μg/ml, than the crude extract with MIC of 12.4 μg/mL against *T*. *brucei*. Moreover, FB-3 fraction and calycosin were showed a close cytotoxicity result to diminazen (as its minimum IC_50_ value > 30 μg/ml) against human leukemia cell lines. The results of this study indicate the richness of red Brazillian propolis with phenolic compounds with health benefits which could be incorporated in pharmaceutical and dietary products as source of several nutraceuticals. As this study is limited to isolation of two compounds and two biological effects, further phytochemical and biological investigation for red Brazilian propolis is recommended in the future studies to isolate bioactive metabolites.

## Supporting information

S1 File (PDF)
