# Supplementary material for: Evaluation of the antitrypanosomal activity, cytotoxicity and phytochemistry of red Brazilian propolis
Source: PLoS One. 2024 Nov 19;19(11):e0313987. doi: 10.1371/journal.pone.0313987 (PMC11575780; doi:10.1371/journal.pone.0313987)
Supplement: S1 File — (PDF) [file pone.0313987.s001.pdf]

Supplementary Materials

Evaluation of the antitrypanosomal activity, cytotoxicity and phytochemistry of Red Brazilian propolis

Table S1: The most abundant compounds in red Brazilian's fraction FB-3 when analyzed by reversed phase LC-MS in negative ion mode.

Peak No	RT (min)	$[M\text{-}H]^-$	Chemical formula	(ppm)	Intensity	Compound Name	Class
1	13.05	271.06	$C_{15}H_{11}O_5$	1.525	E 7	Luteolinidin	Anthocyanins
2	14.58	283.06	$C_{16}H_{11}O_5$	0.824	E 7	calycosin	Isoflavone
3	16.08	255.07	$C_{15}H_{11}O_4$	1.129	E 6	liquiritigenin	Flavonoid
4	28.78	285.11	$C_{17}H_{17}O_4$	0.378	E 5	Unknown phenolic	Phenolic
5	34.95	269.08	$C_{16}H_{13}O_4$	0.921	E 6	7-methoxy Apigeninidin	Isoflavone
6	36.16	507.24	$C_{30}H_{35}O_7$	0.677	E 7	Unknown phenolic	Phenolic
7	41.71	271.1	$C_{16}H_{15}O_4$	0.287	E 6	Unknown phenolic	Phenolic

**Table S2:** Sequence of column chromatography solvent systems and fractions collected.						
**No.**	**He %**	**EtOAc %**	**MeOH %**	**M.P (ml)**	**Fractions obtained**	**Weight (mg)**
1	80	20	0	200	fraction FB1 (M1+M2+M3+M4)	55 mg
2	60	40	0	200	fraction FB2 (M5+M6+M7+M8)	215 mg
3	40	60	0	200	fraction FB3 (M9+M10+M11+M12)	470 mg
4	20	80	0	100	fraction FB4 (M13+M14)	95 mg
5	20	80	0	100	fraction FB5 (M15+M16)	102 mg
6	0	100	0	100	fraction FB6 (M17+M18)	230 mg
7	0	100	0	100	fraction FB7 (M19+M20)	140 mg
8	0	70	30	200	fraction FB8 (M21+M22+M23+M24)	80 mg
9	0	50	50	200	fraction FB9 (M25+M26+M27+M28)	105 mg

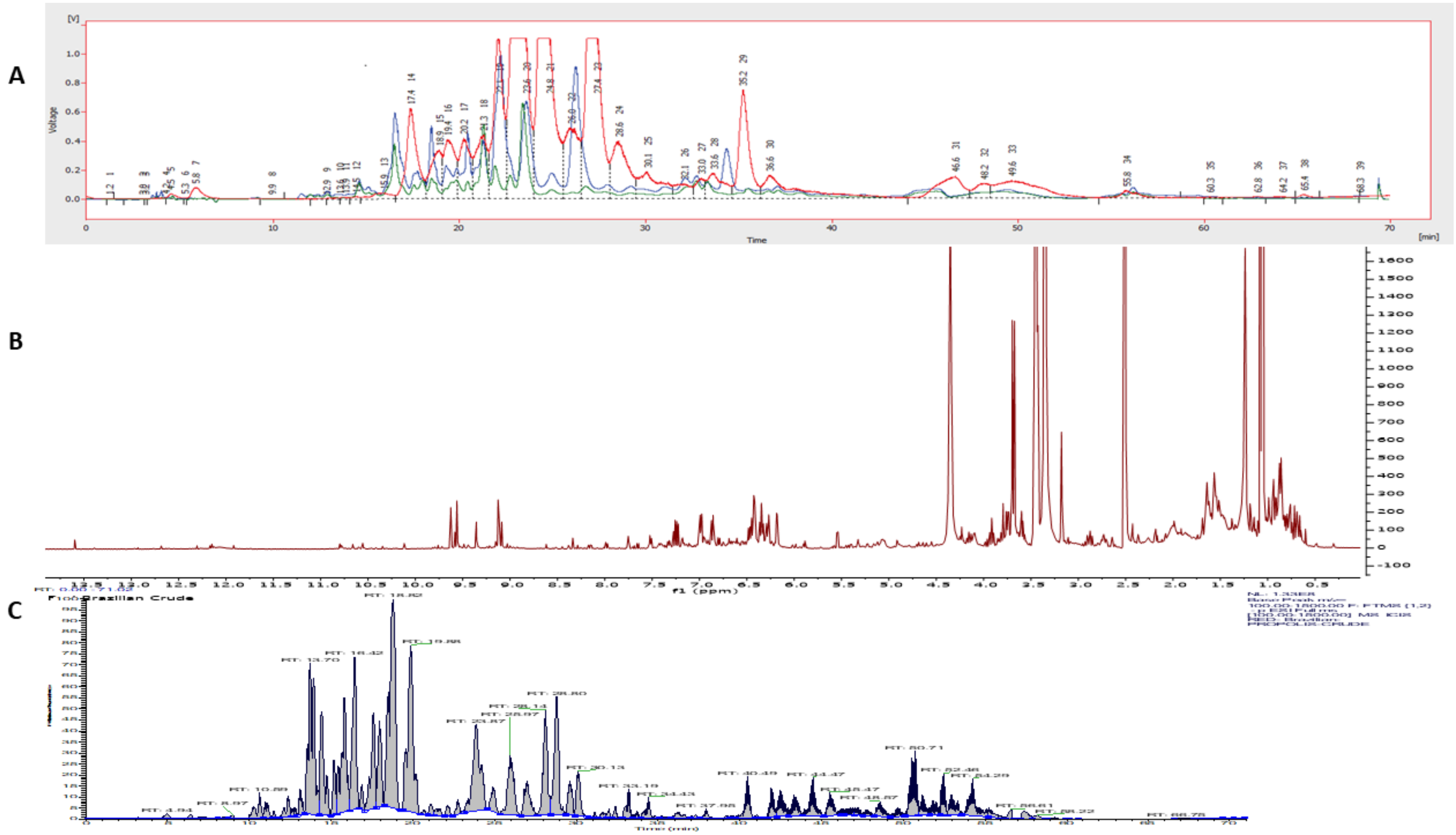

Fig S1: A) Chromatogram of ethanolic extract of Brazilian red propolis by using ELSD-UV. **B)** ^{1}H (400 MHz) NMR spectra of the ethanolic extract of red Brazilian propolis in DMSO-d_6. The main constituents highlighted by ^{1}H NMR spectrum were flavonoids and phenolics, while terpenoids and fatty acids of lesser intensity compared to flavonoids and phenolics were detected as well. MeOH extract was observed to contain aromatic compounds, this was shown by several signals from 6 to 8 ppm as well as phenolic hydroxyl group between 10-13 ppm. **C)** Chromatogram view of ethanolic extract of red Brazilian propolisby LC-MS in negative ion mode (-ve ESI).

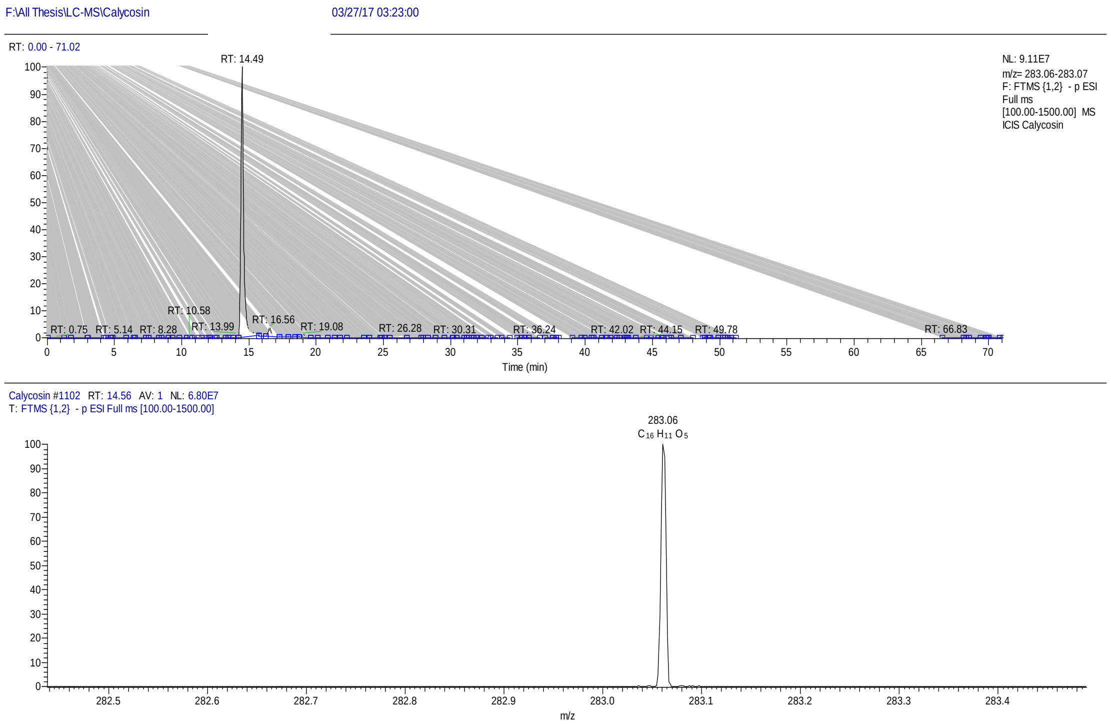

Fig S2: (A) Extracted ion chromatogram corresponding to the mass of calycosin in the negative ion mode (-ve ESI) (B) The spectrum corresponding to the calycosin chromatogram.

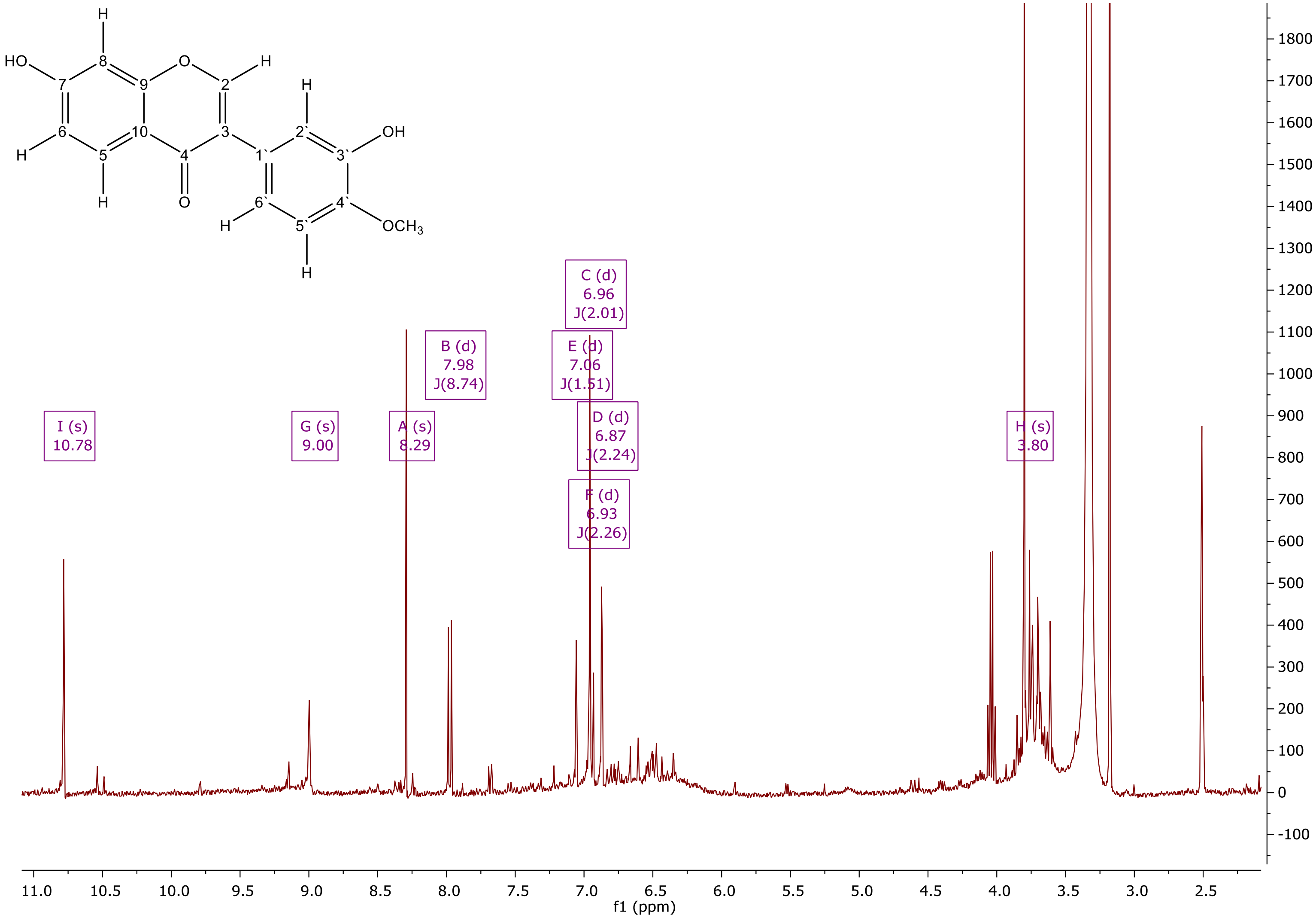

Fig S3: ^{1}H NMR spectrum (400 MHz) of calycosin (FB-3-10) in DMSO-d6

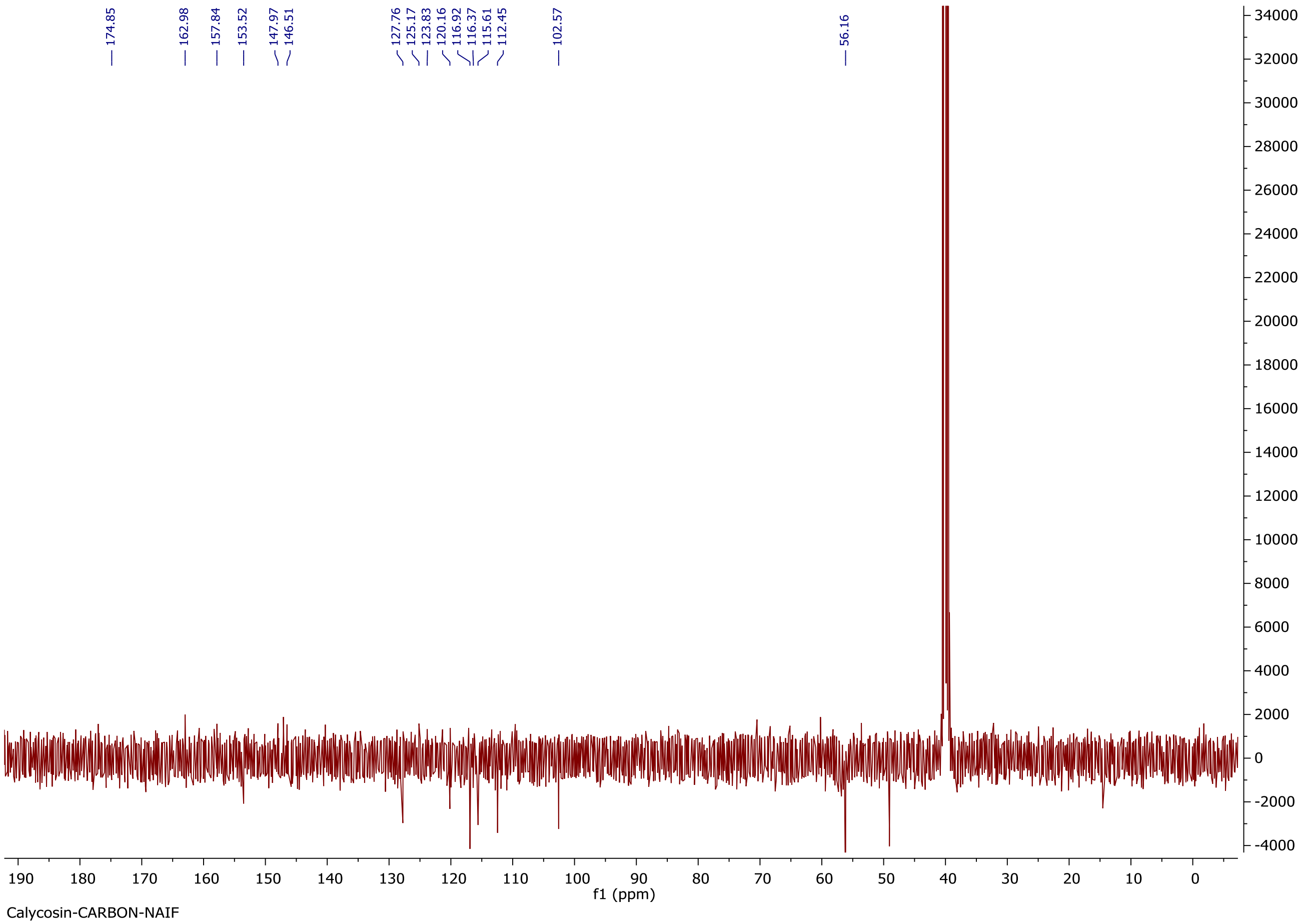

Fig S4: ^{13}C NMR spectrum (100 MHz) of calycosin (FB-3-10) in DMSO-*d6*

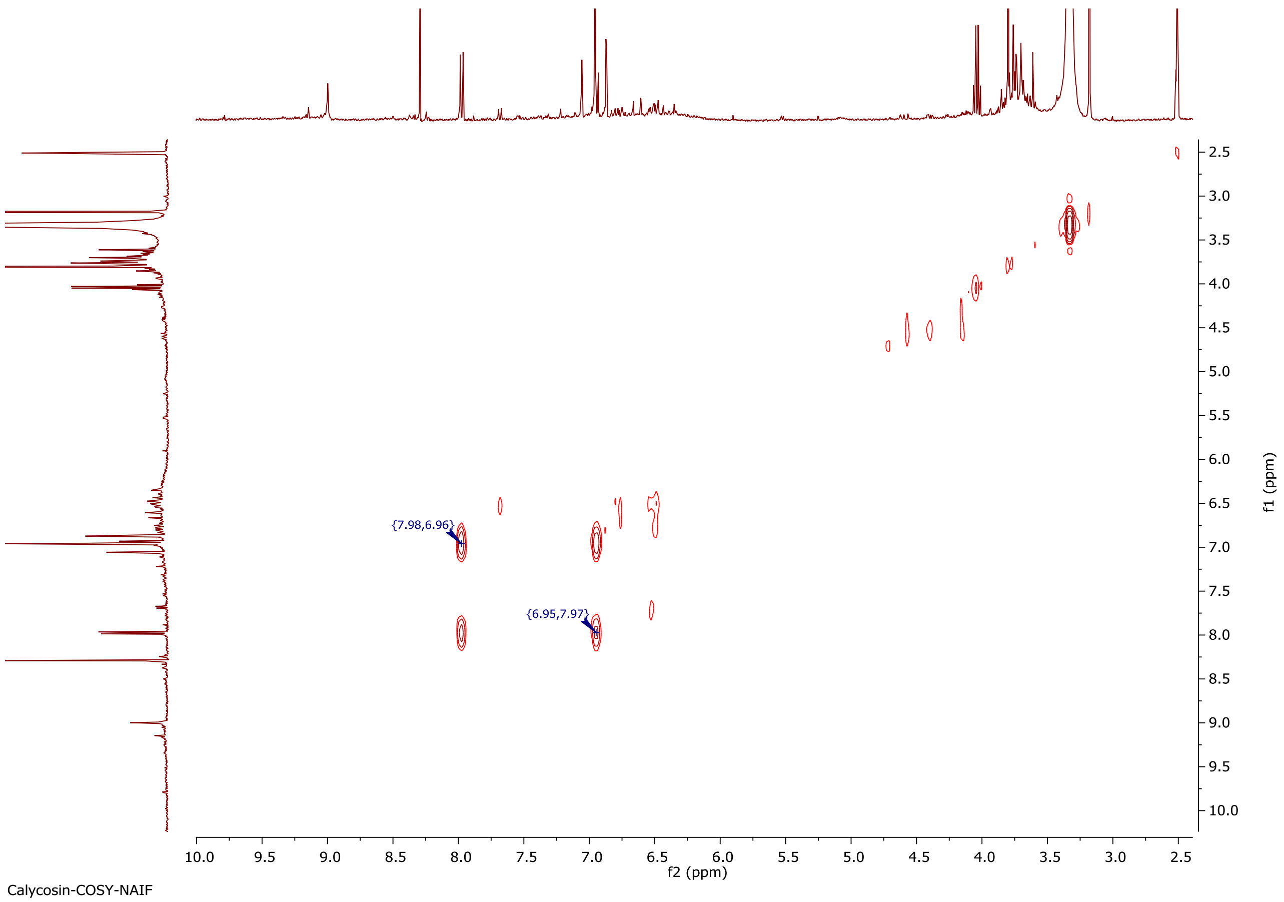

Fig S5: COSY spectrum (400 MHz) of calycosin (FB-3-10) in DMSO-d_6

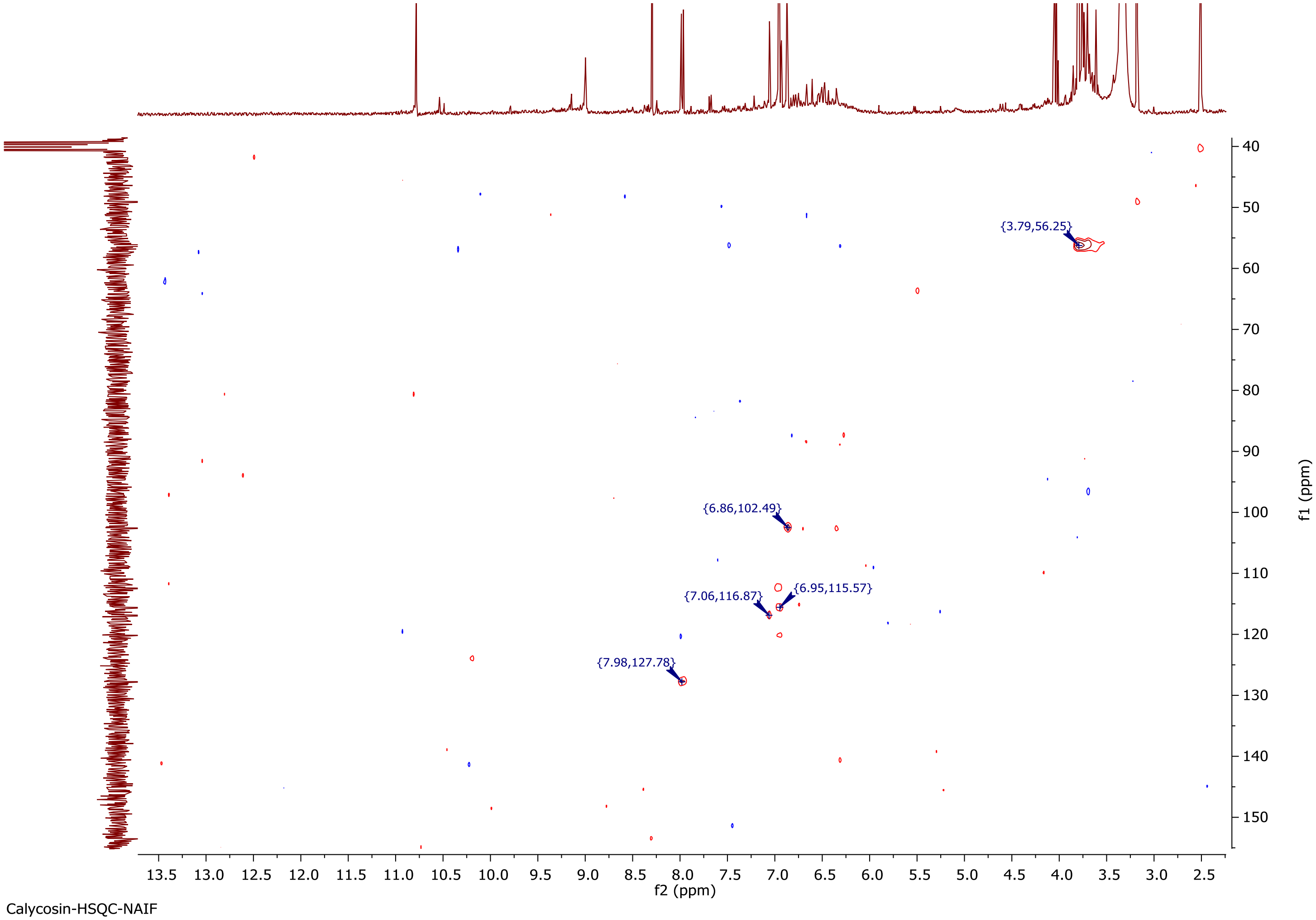

Fig S6: HSQC spectrum (400 MHz) of calycosin (FB-3-10) in DMSO-d_6

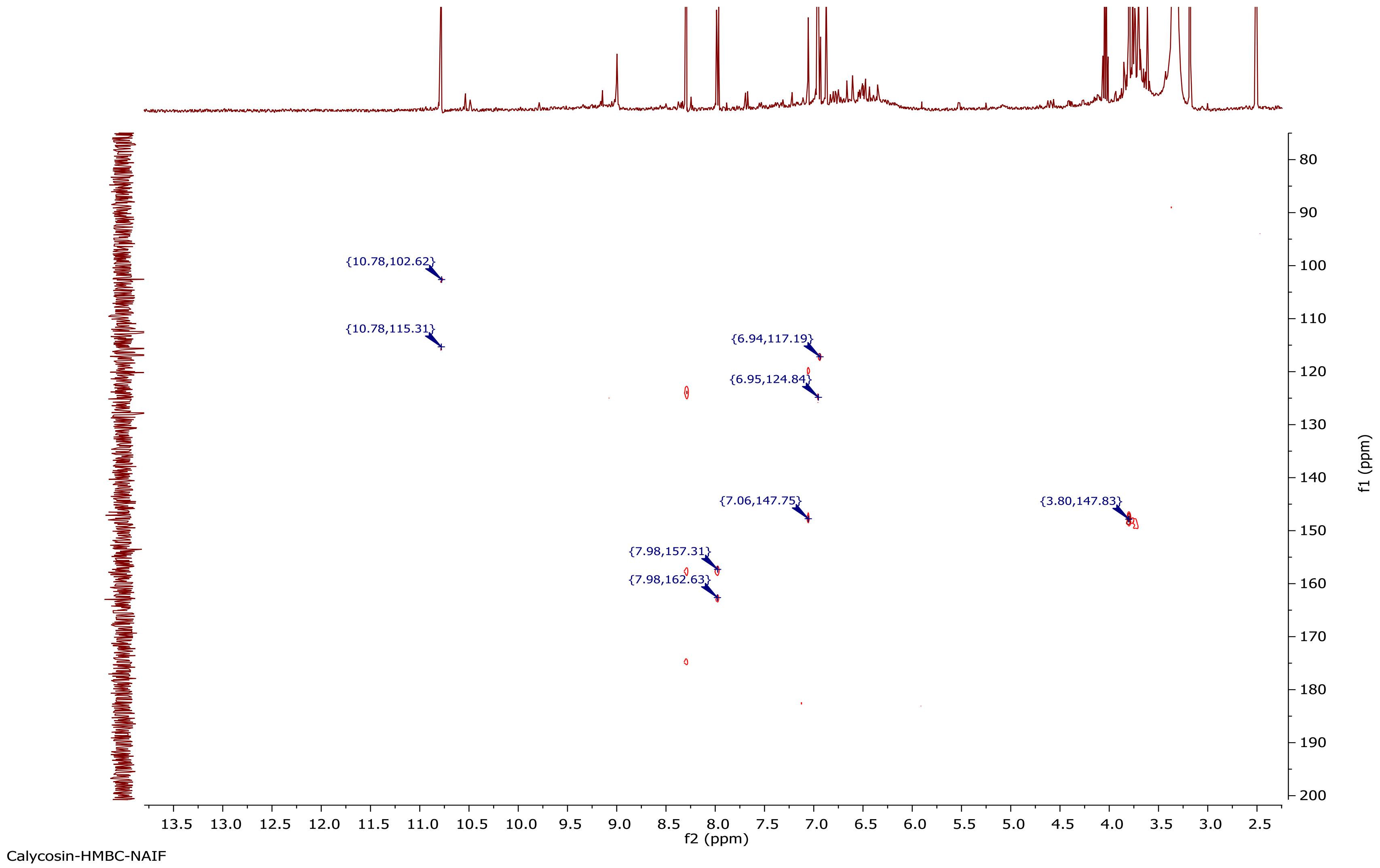

Fig S7: HMBC spectrum (400 MHz) of calycosin (FB-3-10) in DMSO-d_6

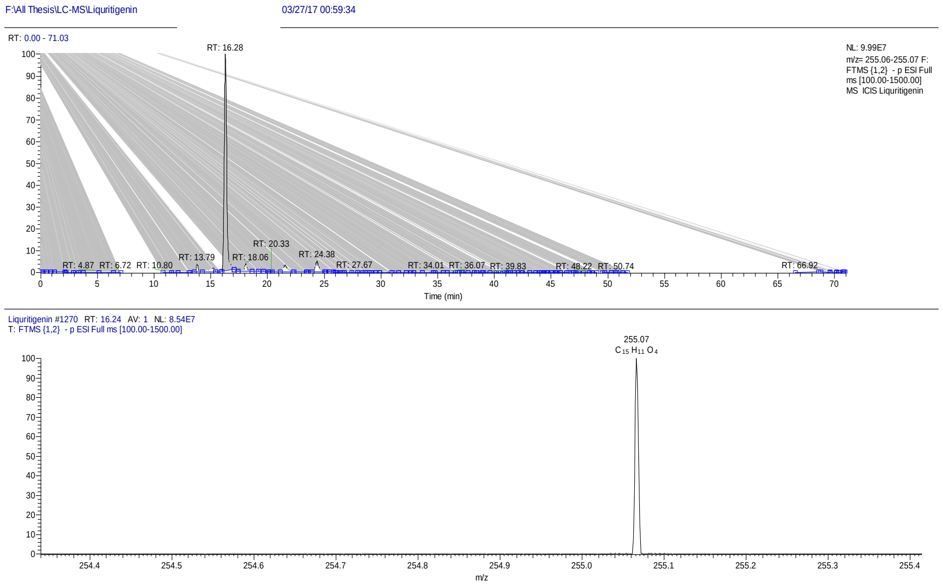

Fig S8: (A) Extracted ion chromatogram corresponding to the mass of liquiritigenin in the negative ion mode (-ve ESI) (B) The spectrum corresponding to the liquiritigenin chromatogram

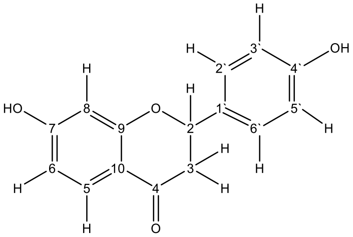

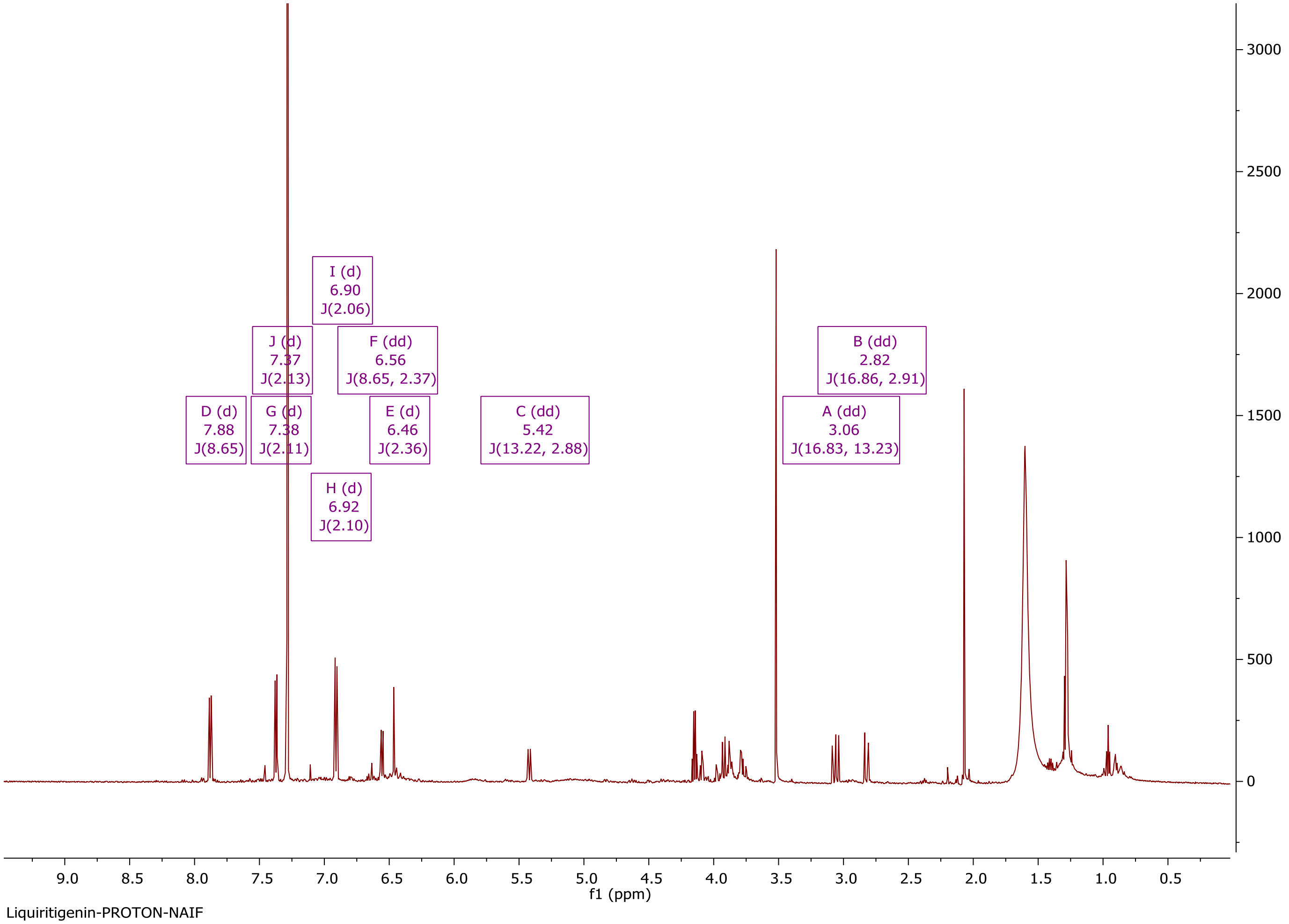

Fig S9: ^{1}H NMR spectrum (400 MHz) of liquiritigenin (FB-3-14) in $CDCl_3$

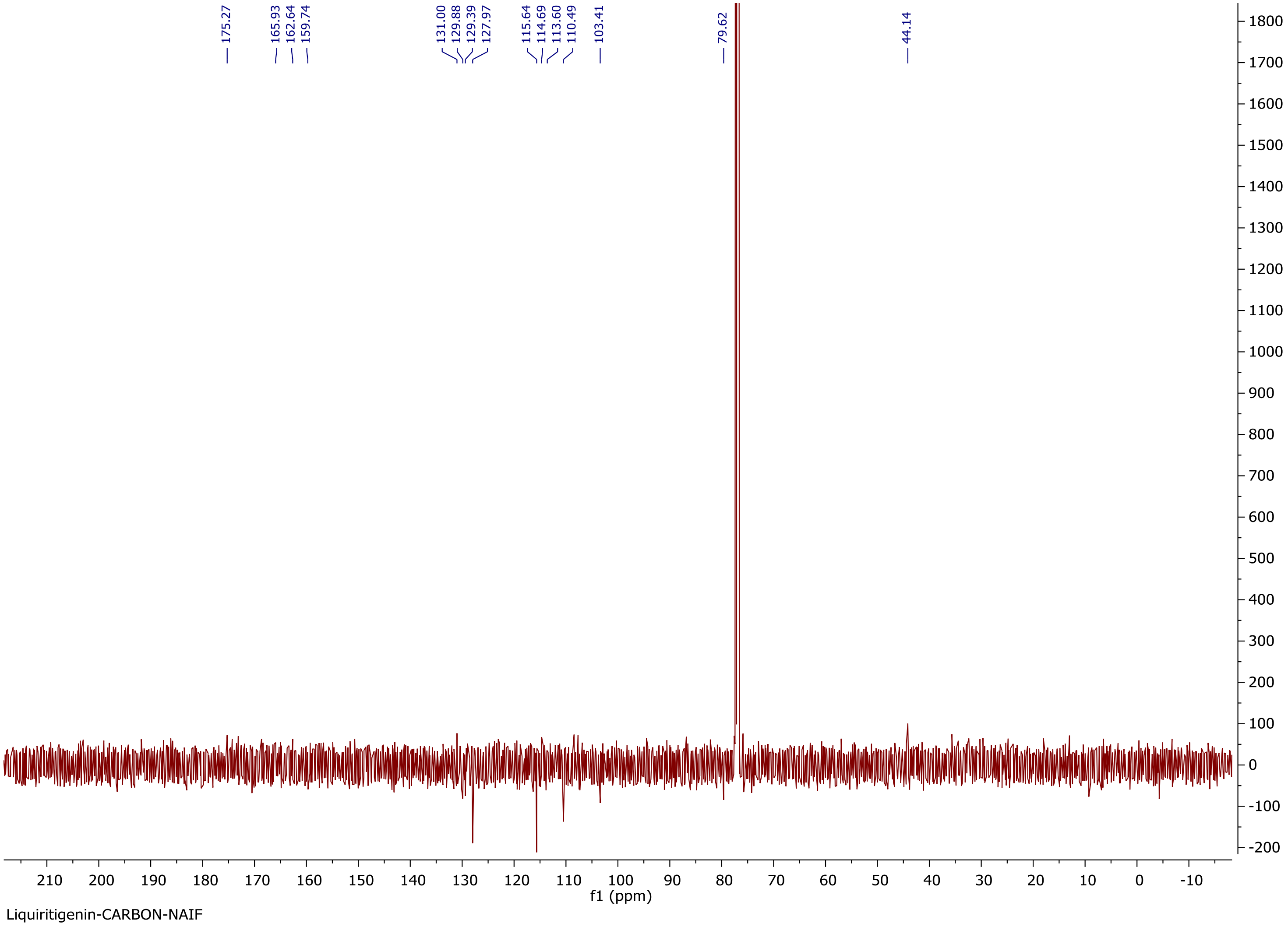

Fig S10: ^{13}C NMR spectrum (100 MHz) of liquiritigenin (FB-3-14) in $CDCl_3$

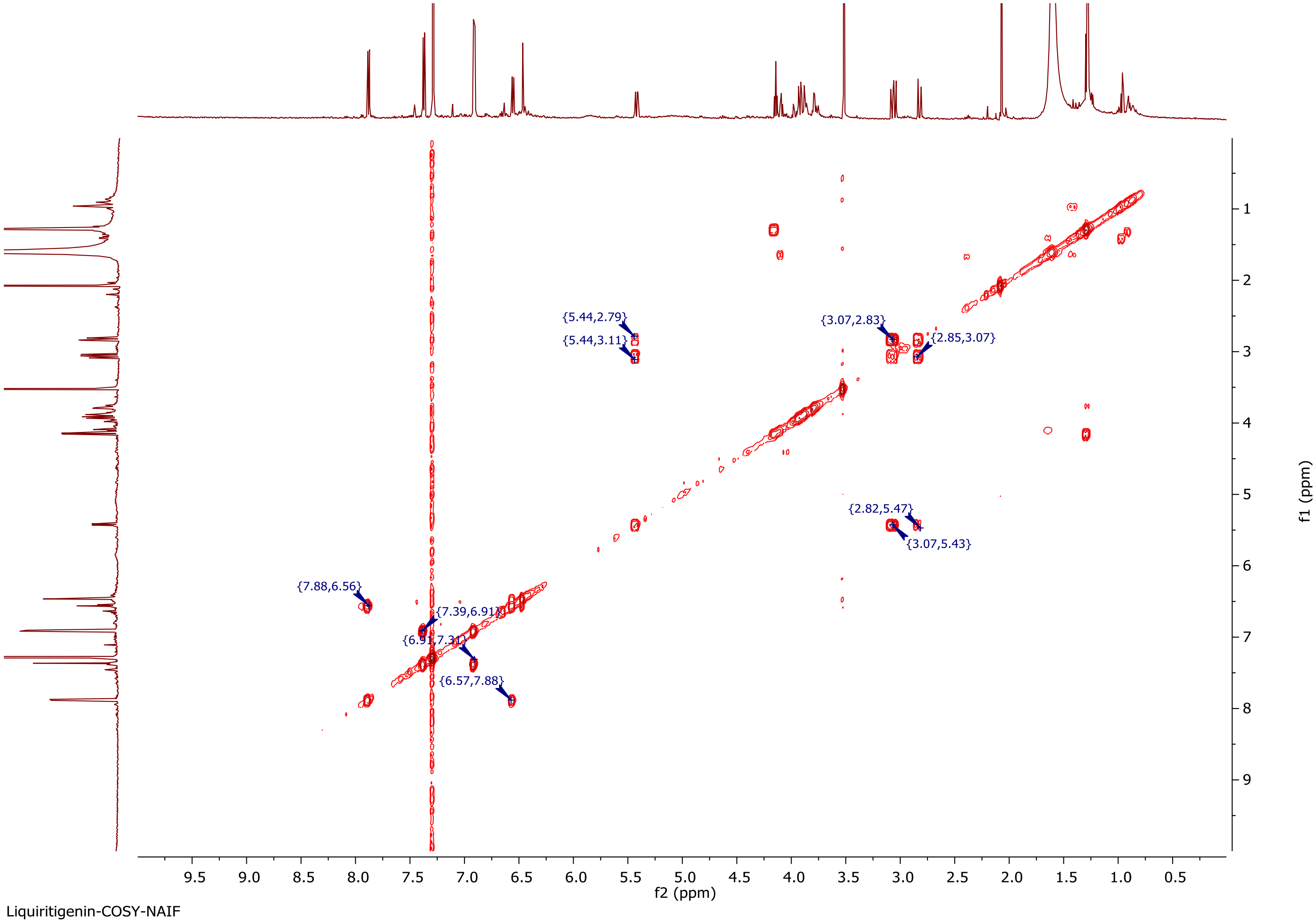

Fig S11: COSY spectrum (400 MHz) of liquiritigenin (FB-3-14) in $CDCl_3$

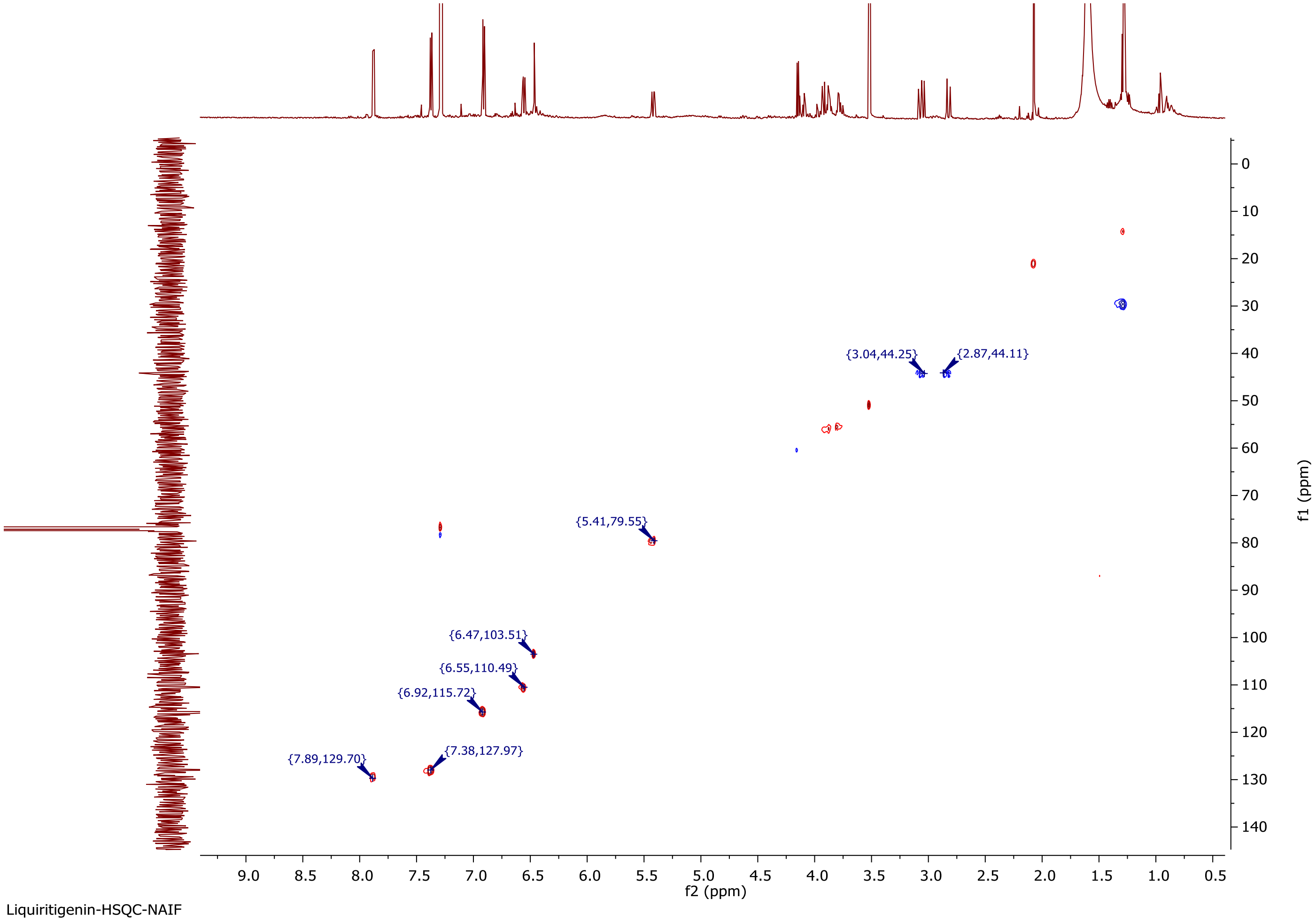

Fig S12: HSQC spectrum (400 MHz) of liquiritigenin (FB-3-14) in $CDCl_3$

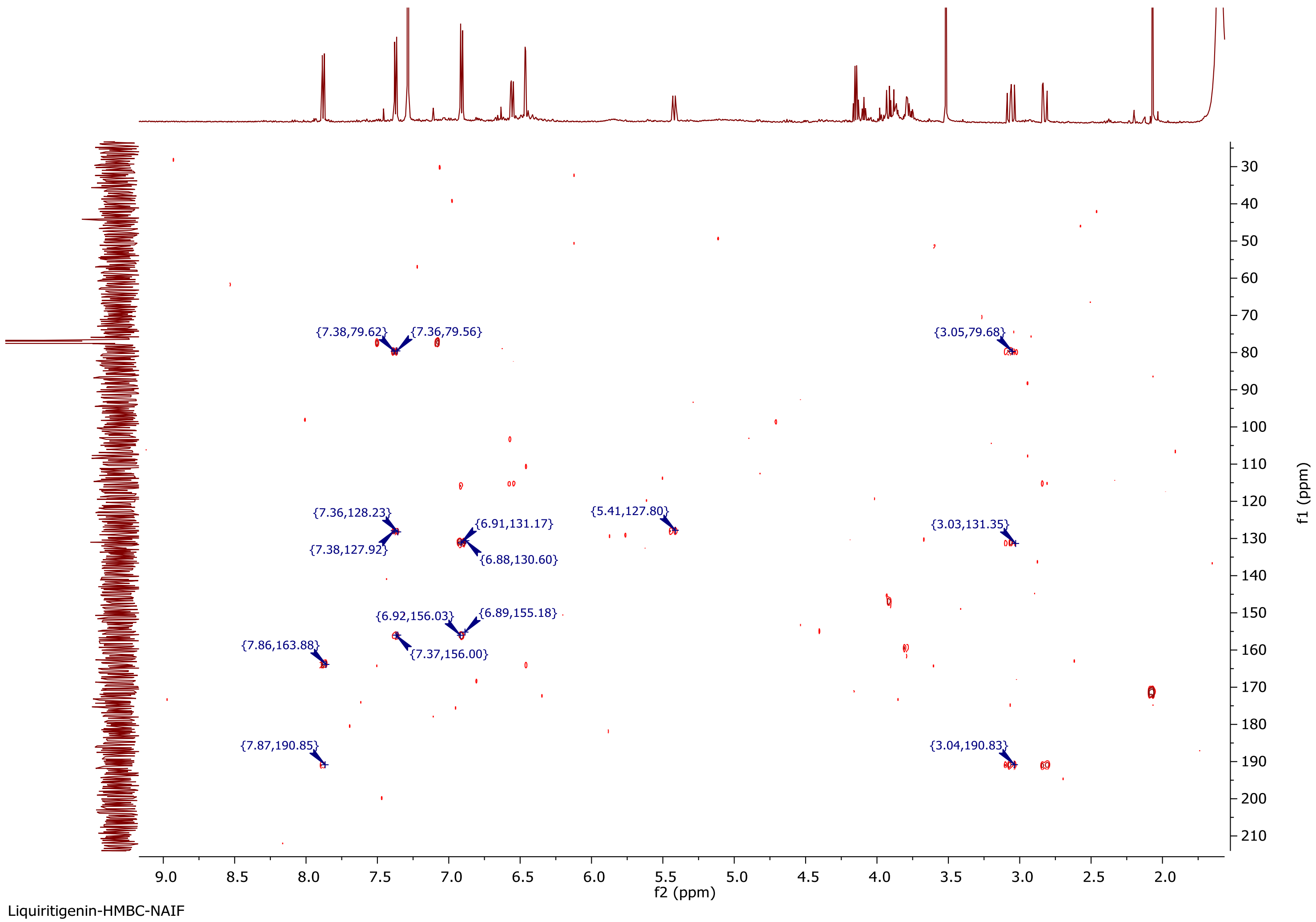

Fig S13: HMBC spectrum (400 MHz) of liquiritigenin (FB-3-14) in $CDCl_3$

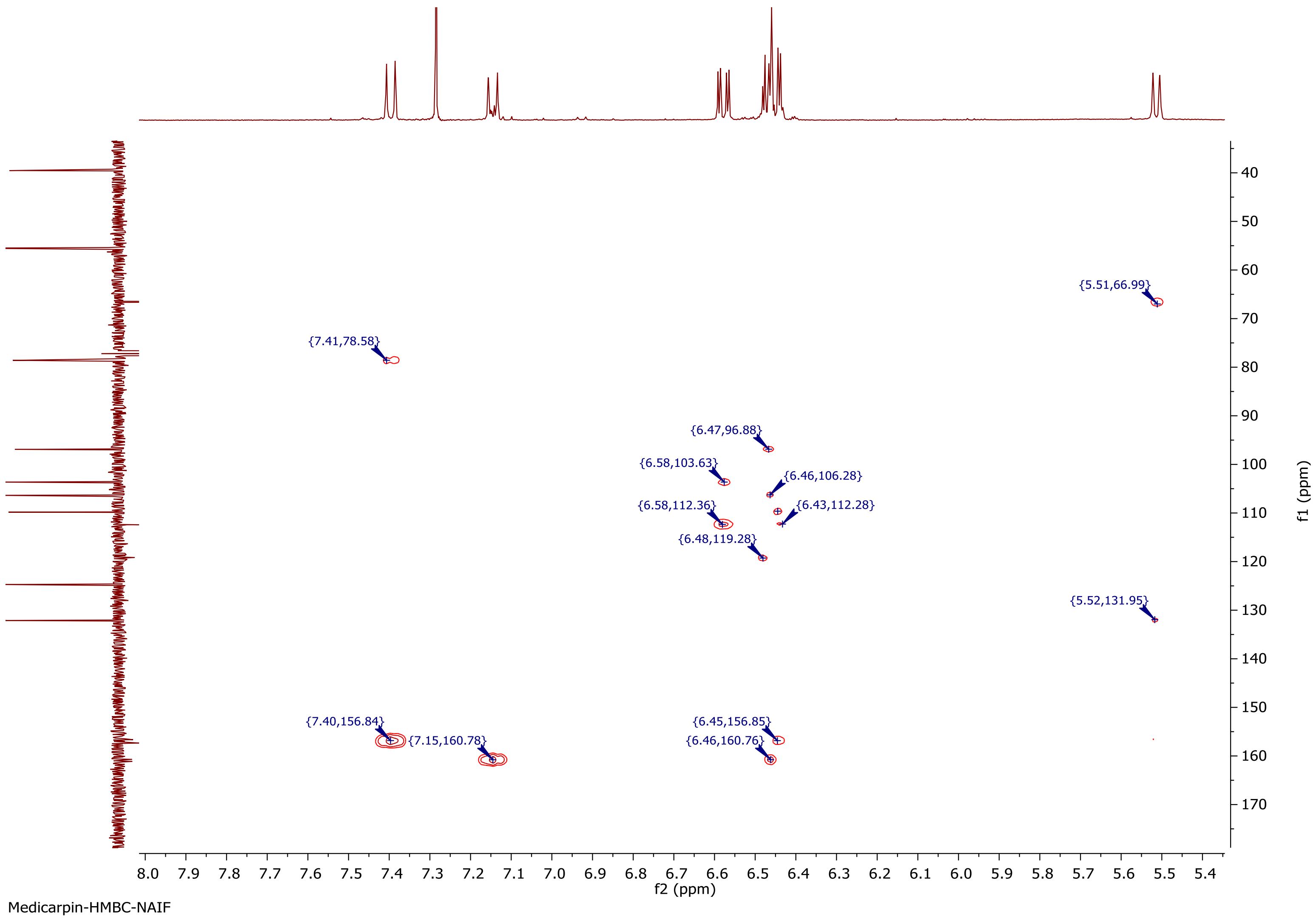

Fig S14: Selected HMBC expansion for the aromatic region of liquiritigenin (FB-3-14) in $CDCl_3$
